# Predicting GD2 expression across cancer types by the integration of pathway topology and transcriptome data

**DOI:** 10.3389/fbinf.2025.1705930

**Published:** 2025-12-04

**Authors:** Arsenij Ustjanzew, Federico Marini, Saskia Wagner, Arthur Wingerter, Roger Sandhoff, Jörg Faber, Claudia Paret

**Affiliations:** 1 Institute of Medical Biostatistics, Epidemiology and Informatics (IMBEI), University Medical Center of the Johannes Gutenberg-University Mainz, Mainz, Germany; 2 Institute for Quantitative and Computational Biosciences (IQCB), Johannes Gutenberg-University Mainz, Mainz, Germany; 3 University Cancer Center (UCT), University Medical Center of the Johannes Gutenberg-University Mainz, Mainz, Germany; 4 Research Center for Immunotherapy (FZI), Mainz, Germany; 5 Department of Pediatric Hematology/Oncology/Hemostaseology, Center for Pediatric and Adolescent Medicine, University Medical Center of the Johannes Gutenberg-University Mainz, Mainz, Germany; 6 Research Group Lipid Pathobiochemistry, German Cancer Research Center, Heidelberg, Germany; 7 German Cancer Consortium (DKTK), Site Frankfurt/Mainz, Mainz, German Cancer Research Center (DKFZ), Heidelberg, Germany

**Keywords:** GD2 prediction, ganglioside, support vector machine, transcriptome analysis, cancer subtypes, metabolic network, reaction activity score, biomarker

## Abstract

**Background:**

The disialoganglioside GD2 is a key cancer therapy target due to its overexpression in several cancers and limited presence in normal tissues. However, experimental assessment is technically challenging and not routinely available. We developed a computational framework that integrates reaction activity derived from transcriptomic data with the glycosphingolipid biosynthesis pathway to predict GD2 expression.

**Methods:**

We computed Reaction Activity Scores from transcriptomic data and weighted the reactions of a glycosphingolipid metabolic network, refining edge weights with topology-based transition probabilities to account for enzyme promiscuity. Cumulative activities of GD2-promoting and -mitigating reactions served as features in a Support Vector Machine (SVM) to model GD2-associated differences between neuroblastoma and normal tissue. SVM decision values were used as a continuous proxy for GD2 expression. We validated the predicted GD2 scores across independent datasets by comparing them with literature-reported values and flow-cytometric confirmation of a model-predicted high-GD2 tumor. Copy-number alteration (CNA) data were integrated to identify candidate genomic biomarkers of GD2-positive samples.

**Results:**

Our SVM-based GD2 score achieved balanced accuracy of 0.80 with a linear kernel, selected due to reduced overfitting risk and interpretability, while matching the accuracy of more complex kernels. The model transferred reliably across six independent RNA-seq datasets and reproduced known GD2 expression patterns, outperforming a two-gene signature in capturing subtype-specific heterogeneity and avoiding overestimation in normal brain tissue. Pan-cancer analyses revealed heterogeneous GD2 expression in several cancer subtypes. Notably, we experimentally confirmed high GD2 expression in clear cell sarcoma of the kidney, consistent with model predictions. CNA analysis implicated B4GALNT1 amplification as a GD2-promoting factor in dedifferentiated liposarcoma. To facilitate adoption of our approach, we developed GD2Viz, an R package with an interactive Shiny application for score computation, visualization, and analysis of user data.

**Conclusion:**

Our computational framework provides a robust, interpretable, biologically grounded predictor of GD2 expression, offering greater consistency and clinical interpretability over existing gene-based signatures. Importantly, with over 20 GD2-directed trials ongoing, our approach may help prioritize tumor entities with high GD2 levels, delineate candidate patient subgroups, and generate testable hypotheses in underexplored cancers, thereby supporting patient stratification and eligibility screening for clinical trials.

## 1 Introduction

Gangliosides are a group of sialylated glycosphingolipids (GSL) involved in differentiation, cell signaling, adhesion, and neuronal development (Yamashita et al., 1999; Kasahara et al., 2000; Yu et al., 2009). The ganglioside GD2 is of particular importance for targeted cancer therapy due to its functional involvement in developmental processes and its pathophysiological roles in cancer diseases (Machy et al., 2023). The expression of GD2 is limited in normal tissues (NT), particularly described in the central nervous system and peripheral sensory nerve fibers (Machy et al., 2023). Conversely, GD2 is overexpressed on the surface of several tumor types, including neuroblastoma (NB), melanoma, small-cell lung cancer, and various sarcomas (Nazha et al., 2020; Machy et al., 2023). This makes GD2 a promising target for therapeutic interventions, such as monoclonal antibodies and CAR-T cell therapies (Richards et al., 2018; Das et al., 2024; Testa et al., 2024). Among the aforementioned cancers, neuroblastoma exhibits particularly high GD2 levels, and anti-GD2 antibodies are already part of the standard treatment for high-risk NB (Chan and Chan, 2022). Currently, 23 clinical studies listed on ClinicalTrials.gov are recruiting patients across multiple tumor types for GD2-targeted therapies (as of 2025-02-06). However, clinical outcomes in tumor entities beyond neuroblastoma have been variable (Gargett et al., 2024; Kaczanowska et al., 2024), underscoring the critical need for precise quantification of GD2 expression to enable optimal patient stratification and maximize the efficacy of anti-GD2 therapeutic interventions.

At present, the assessment of GD2 expression in tumors is primarily performed using immunohistochemistry (IHC) on formalin-fixed, paraffin-embedded (FFPE) tissue samples (Nazha et al., 2020). However, this approach is inherently limited because lipid antigens like GD2 are extracted during the solvent-based tissue processing, leading to unreliable detection (Vos et al., 2019). As a result, no IHC-based diagnostic assays for GD2 are currently available on the market. Alternative methods such as liquid chromatography-tandem mass spectrometry (LC-MS/MS) and flow cytometry show promise for sensitive and quantitative detection of GD2, but these techniques have yet to be fully validated or standardized for routine clinical use (Paret et al., 2022). Consequently, these methodologies are not readily available for routine clinical applications, particularly in resource-limited settings; moreover, no diagnostic assays are available on the market to detect GD2.

RNA sequencing (RNA-seq) has emerged as a powerful tool in the field of clinical oncology, offering a comprehensive view of gene expression. RNA-seq data are increasingly integrated into precision oncology programs designed to create a direct link between molecular diagnostics and prospective clinical trials (Lier et al., 2018; Berlanga et al., 2022). These programs leverage RNA-seq to comprehensively profile tumor biology, enabling the identification of actionable molecular targets and biomarkers (Seidmann et al., 2025). RNA-seq could present an opportunity to assess ganglioside-related gene activity and could be used to infer GD2 levels. However, translating expression values from transcriptomic data into accurate ganglioside phenotype predictions remains a challenge, underscoring the need for methods and models that bridge the gap between gene expression and metabolic profiles.

**FIGURE 1:**
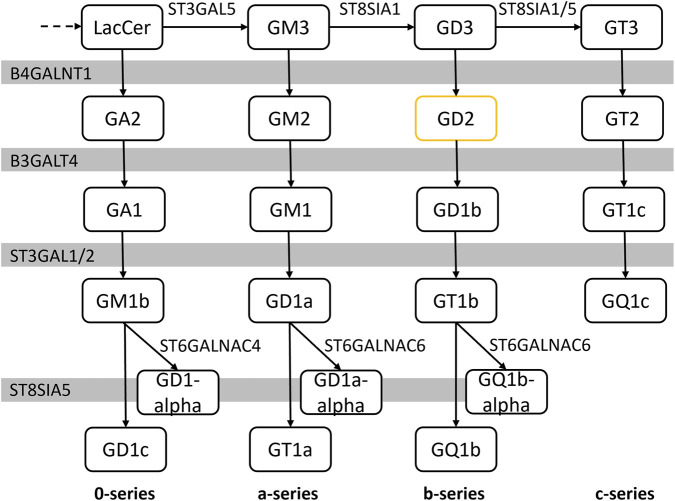
The ganglioside biosynthesis pathway is a sequential process and is composed of four parallel series. The enzymes of these series encoded by *B4GALNT1*, *B3GALNT4*, *ST3GAL1/2* and *ST8SIA1* have a broad specificity to its substrates and are therefore involved across the series. (LacCer: lactosyl ceramide). The figure is based on the latest version of the KEGG pathway 00604 as of 2022-04-13.

Previous methods for predicting GD2 expression have primarily relied on individual genes or small gene signatures (Ruan et al., 1999; Mabe et al., 2022). For example, Sorokov et al. (2020) proposed a two-gene signature comprising *ST8SIA1* and *B4GALNT1*, aiming to correlate their expression with a GD2-positive phenotype (Sorokin et al., 2020). Similarly, Sha et al. (2021) developed a glycosyltransferase score based on neuroblastoma datasets, which correlated with GD2 positivity (Sha et al., 2021). However, these models are limited by their narrow focus on single genes or cancer types, neglecting the broader metabolic pathway and enzyme-substrate specificities involved in ganglioside metabolism: The ganglioside biosynthesis pathway is a stepwise process that involves multiple enzymes, beginning with the formation of glycosylceramide from ceramide by the enzyme encoded by *UGCG* and continuing with the sequential addition of saccharides through a variety of glycosyl- and sialyltransferases (Figure 1). The initial enzymes (encoded by *ST3GAL5* and *ST8SIA1*) in this pathway demonstrate high substrate specificity, directing the formation of distinct ganglioside series (0-, a-, b-, and c-series), while downstream enzymes, such as those encoded by *B4GALNT1* and *B3GALT4*, exhibit broader substrate specificity and elongate all four series (Sandhoff and Sandhoff, 2018; Sandhoff et al., 2018). Importantly, the aforementioned models do not take into account that GD2 is an intermediate in the biosynthesis of more complex gangliosides present in normal tissues. Therefore, downstream enzymatic reactions that convert GD2 into subsequent gangliosides and the ambiguous enzyme-substrate specificities across the ganglioside series should also be considered when evaluating models for predicting GD2 expression.

In this study, we hypothesized that the integration of gene expression data with ganglioside biosynthesis pathway information may serve as an effective predictor of the GD2-positive phenotype. Our central premise was that the enzymatic activity within this pathway, particularly the balance between GD2-promoting and GD2-mitigating reactions, could be modeled to reflect GD2 expression as a biological continuum, rather than a binary state. NB and NT RNA-seq samples were chosen to represent the full range of GD2 expression from high GD2 levels commonly found in NB to low or absent expression in NT. To map gene expression to reactions of the ganglioside metabolism pathway, we computed Reaction Activity Scores (RAS) and addressed the ambiguity regarding the substrate specificity of certain enzymes by adjusting the RAS values with the transition probability of the pathway topology (Ustjanzew et al., 2024). These RAS values of GD2-promoting and -mitigating reactions were then utilized to train a machine learning model to infer GD2 levels. Finally, we developed the R package GD2Viz, which is available at https://github.com/arsenij-ust/GD2Viz, to provide scientists and clinicians an easier access to our methodology. The package contains R functions that facilitate the prediction of the GD2 score, encapsulated in an interactive Shiny app that enables users to estimate and visualize GD2 scores from their own datasets.

In conclusion, we present a computational approach capable of predicting GD2 molecular phenotypes across diverse cancers, along with the GD2Viz tool, which can assist in diagnostic decisions and provide deeper insights into the regulation of GD2 in cancer biology.

## 2 Materials and methods

### 2.1 Workflow overview

**FIGURE 2:**
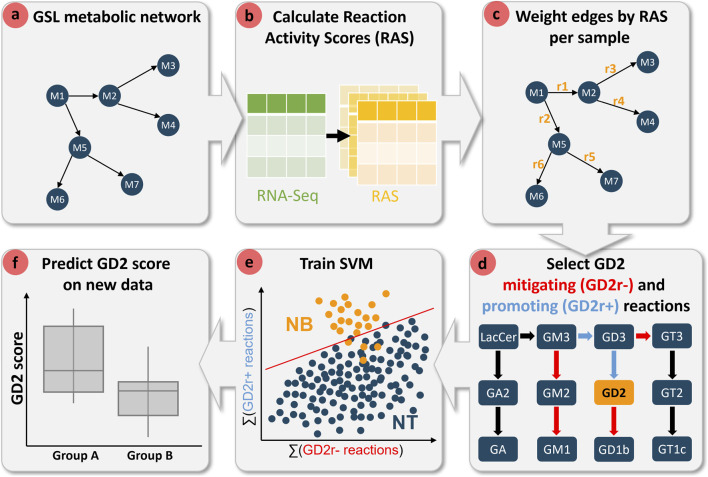
Workflow diagram of the computational pipeline for predicting GD2 scores using transcriptome data and metabolic network topology. **(a)** A glycosphingolipid (GSL) metabolic network is constructed, where nodes represent metabolites and edges denote biochemical reactions. **(b)** Reaction Activity Scores (RAS) are computed from RNA-Seq data to quantify reaction activities across samples. **(c)** The metabolic network is weighted based on RAS values for each sample. **(d)** Reactions are categorized as either mitigating (GD2r-, red) or promoting (GD2r+, blue) based on their position in the metabolic pathway in respect to GD2. **(e)** A support vector machine (SVM) classifier is trained using the cumulative activity of GD2r- and GD2r+ reactions to distinguish neuroblastoma (NB) from normal tissue (NT) samples. **(f)** The trained model is then applied to predict GD2 scores in independent datasets, facilitating group comparisons.

Figure 2 provides a schematic overview of the computational pipeline for model development and GD2 score prediction. The workflow begins with the construction of a GSL metabolic network by merging four Kyoto Encyclopedia of Genes and Genomes (KEGG) pathways related to sphingolipid metabolism (see Section 2.3 for further details). In this metabolite-centric network, metabolites are represented as nodes, while biochemical reactions define edges. Next, Reaction Activity Scores (RAS) are derived from RNA-seq data to quantify reaction activities across individual samples. These scores are subsequently used to assign weights to the metabolic network edges, enabling sample-specific profiling on the reaction-level rather than gene-level.

To refine reaction-level resolution, we incorporated network topology by integrating node transition probability with RAS values. This approach facilitates the disambiguation of enzymatic substrate specificity. In this study, we systematically assessed the impact of raw and topology-adjusted RAS values on the computed GD2 score. Reactions were labeled based on their influence on GD2 levels as mitigating (GD2r-) and promoting (GD2r+) reactions, according to their position in the metabolic pathway.

An SVM classifier was trained using the cumulative activity of the GD2r+ and GD2r- reactions to distinguish NB from NT samples. Various sets of GD2r+ and GD2r- reactions, as well as different SVM kernels, were evaluated to optimize model performance and its explainability.

Finally, the trained model was applied to predict GD2 scores in independent datasets, which were used for subsequent validation based on expected biological outcomes and existing literature. The GD2 score enables comparative analyses across different biological groups, such as cancer subtypes, and may further serve as a basis for the identification of possible biomarkers and cancer-associated genes linked to GD2 expression. The workflow was executed fully in R (version 4.1.3).

### 2.2 Data acquisition and preprocessing

To model GD2 expression along a molecular continuum, we used neuroblastoma (NB) and normal tissue (NT) samples from the UCSC Toil RNA-seq recompute compendium (TCGA-TARGET-GTEx cohort, n = 19,109). After filtering and annotation, the training dataset comprised 7,548 samples, including 136 NB and 7,412 NT samples (see Table 1 for sample numbers and dataset overview). Gene expression data were normalized using the median-of-ratios method from the DESeq2 package, followed by log10 transformation.

**TABLE 1:** Datasets used for training and model validation.

Dataset	# Samples	References
Training datasets:		
TARGET NB	136	Pugh et al. (2013)
GTEx	7,845	GTEx Consortium (2013)
Evaluation datasets:		
TCGA	10,529	Colaprico et al. (2016), Lehmann et al. (2016)
TARGET	734	Pugh et al. (2013)
St. Jude Cloud	2,853	Downing et al. (2012), Newman et al. (2021), Rusch et al. (2018), Schwartz et al. (2021)
CBTTC	970	Ijaz et al. (2020)
GSE117446	29	Krug et al. (2019)
GSE147635	15	Weiss et al. (2021)
GSE180514	8	Mabe et al. (2022)

For model validation and subtype exploration, GD2 scores were predicted in multiple independent RNA-seq datasets (Table 1). Full preprocessing steps and dataset descriptions are provided in the Supplementary Material and Figures, Section 1.1.

### 2.3 Graph construction

A metabolite-centric graph was constructed using the R packages *NetPathMiner* (v1.30.0) (Mohamed et al., 2014) and *igraph* (v1.4.2) using data from KEGG. Nodes represent metabolites, and edges connect metabolites involved in the same reactions, with edge metadata specifying the responsible enzymes. The graph includes four KEGG pathways (hsa00600, hsa00601, hsa00603, hsa00604) covering sphingolipid and glycosphingolipid metabolism, including the lacto-, neolacto-, globo-, and ganglio-series. Degradation reactions R06010 (GM1 to GM2) and R06004 (GM2 to GM3) were excluded as they are not competing enzymatic reactions of the biosynthesis, resulting in a metabolic graph containing in total of 104 nodes and 116 edges. KEGG data was retrieved on 2022-07-07.

### 2.4 Weighting of the graph

In the following section, we provide a brief overview of the methodology introduced in Ustjanzew et al. (2024) to integrate the pathway topology with transcriptomic data (Ustjanzew et al., 2024). For each sample, the metabolic graph was weighted using RAS and integrated with transition probabilities. The weighting of the metabolic graph is initiated by the computation of RAS values based on gene expression levels and gene-protein-reaction (GPR) rules, which serve as a proxy for the activity of enzymatic reactions. In order to account for the lack of enzyme specificity in the ganglioside metabolism pathway, the RAS values were adjusted by a topological metric, namely the RAS-based transition probabilities of the metabolic graph. For a more detailed explanation of the RAS adjustment methods, please refer to Ustjanzew et al. (2024).

#### 2.4.1 Calculating reaction activity scores

The edges in the metabolic graph were assigned weights based on RAS, a measure of enzymatic activity for each reaction in the graph, as introduced by Graudenzi et al. (2018). The RAS was calculated based on the decimal normalized gene expression levels from patient samples and the GPR association rules derived from the *Homo sapiens* genome-scale metabolic model (Human1) (Robinson et al., 2020). GPRs are logical formulas that describe the enzymes involved in specific reactions and how they relate to one another when multiple enzymes are associated with a common reaction. The minimum gene expression levels were assigned to reactions with an “AND” operator (e.g., multiple subunits), while those with an “OR” operator (e.g., isoenzymes) got the sum of gene expression levels across the involved genes.

#### 2.4.2 Adjusting RAS by transition probabilities

For each sample, we calculated a transition probability (TP) matrix, representing the likelihood of transitioning from one node to the next in a single step in the metabolic graph based on the RAS values of outgoing edges. The TP for an outgoing edge of node x was calculated by dividing the edge’s RAS value by the sum of all outgoing edges for that node. The edge weights were adjusted by multiplying the TPs by the RAS values:

$$P\left(e\right)=\frac{r_{e}}{\sum \varepsilon \left(x\right)},$$

Where $P\left(e\right)$ is the TP of edge e = (x → y), $r_{e}$ denotes the RAS of edge e , and $\varepsilon \left(x\right)$ is the set of outgoing edges from node x. Edge weights are then TP-adjusted as:

$$r_{e}^{\left(TP\right)}=r_{e\;}\times P\left(e\right),$$

so that promiscuous enzymes contributing to multiple competing reactions have their influence partitioned across branches in proportion to the local probability of proceeding along each branch.

In pathway segments that contain long linear stretches (single outgoing edge at each step), particularly along the 0-, a-, b-, and c-series within the ganglioside metabolism pathway, $P\left(e\right)=1$ across those segments and preserves identical values along the series (Supplementary Figures 1a–c). To resolve this, we employed an alternative TP matrix to adjust the RAS values. In this alternative adjustment method (hereafter, we will refer to this method as the recursive adjustment), the TPs that were equal to 1 (due to their status as the sole outgoing edges) were assigned the next available upstream TP that was not equal to 1 (Supplementary Figure 1d). As with the original TP, the alternative TP matrix was multiplied by the RAS values per sample:

$\tilde{P}\left(e\right)=P\left(e_{j}\right)$ for the smallest *j* as the position along the upstream path from the nearest branching node with $P\left(e_{j}\right)\neq 1$ , and the final adjusted weight as:

$$r_{e}^{\left(TP-rec\right)}=r_{e\;}\times \tilde{P}\left(e\right).$$

### 2.5 Model construction & training

**FIGURE 3:**
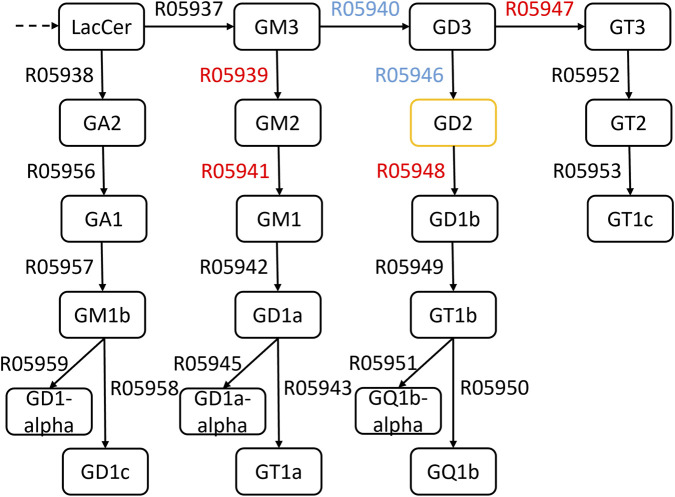
Partial view of the constructed GSL network based on the KEGG-pathway of the glycosphingolipid biosynthesis - ganglio series. *Ab initio*-defined GD2 promoting (GD2r+) reaction IDs are blue and GD2 diminishing (GD2r-) reaction IDs are red.

Starting with raw or TP-adjusted RAS matrices of the training dataset, where columns correspond to reactions and rows to samples, GD2-promoting reactions (GD2r+), namely R05946, R05940, and GD2-mitigating reactions (GD2r-), namely R05939, R05948, R05947, R05941, were selected based on their position in the gangliosphingolipid metabolism pathway (Figure 3). Hereafter, we refer to this selection as *ab initio* reactions. The summed RAS of GD2r+ and GD2r- are later used as two predictor features for an SVM to solve a binary classification task of distinguishing NB samples from NT samples. The predicted decision values (which are equivalent to the sample distance from the SVM hyperplane in case of a linear kernel) are used as a proxy for the GD2 expression level and referred to as the GD2 score.

For model training, we employed the ksvm function from the kernlab package (version 0.9-32) on 136 NB and 7,548 NT samples. In the remainder of this work, we refer to the GD2 score models based on a) RAS values as *ras*, b) based on TP-adjusted RAS values as *rasTP*, and c) based on recursively adjusted RAS values as *rasTPrec*.

### 2.6 Model evaluation

#### 2.6.1 Alternative kernel functions

First, our exploration involved training SVM models with different kernel functions, such as linear, radial basis, polynomial, hyperbolic tangent, Laplace, Bessel, and ANOVA. Each kernel was evaluated across varying hyperparameters to determine its effect on classification performance. The hyperparameter values are reported in Supplementary Table 1.

#### 2.6.2 Evaluation of GD2-promoting and -mitigating reaction sets

To validate whether the *ab initio*-defined GD2r+ and GD2r- predictive variables contribute meaningfully to the classification task, a permutation test was conducted on the training dataset with 10,000 random permutations of GSL reaction labels (two random reaction labels for GD2r+ and four for GD2r-). The null hypothesis was that the *ab initio*-defined reactions of GD2r+ and GD2r- do not have any true predictive relevance. The binary class predictions (NB samples labeled as 1 and NT samples as 0) yielded empirical p-values for different model quality metrics by the caret package (version 6.0-94). Due to the unbalanced nature of the training dataset, we employed four quality metrics to ascertain whether the model exhibited better performance than that of a classifier based on random reactions: balanced accuracy, precision, F1-score, and recall. p-values were computed as the number of permuted metrics with bigger values compared to the observed metric of the *ab initio* model, divided by the total number of permutations.

Additionally, the reactions of GD2r+ and GD2r- were manually altered based on pathway-plausible considerations, resulting in 16 additional reaction sets. We evaluated these reaction sets with the aforementioned quality metric calculation. Precision-recall Receiver Operating Characteristic (PR ROC) curves and area under the curve (AUC) were computed using the PRROC (version 1.4) and ROCR (version 1.0-11) R packages.

#### 2.6.3 Prediction of GD2 scores

To ensure scale consistency and homoscedasticity between the training and independent datasets in the prediction of GD2 scores, the RNA-seq data from the independent datasets must undergo the same normalization and transformation steps as the training dataset (Goksuluk et al., 2019). As the DESeq2 median of ratios method was employed for the normalization of the training dataset, the size factors (*s* ^*^) of the new datasets (*x* ^*^) should be estimated according to the following formula:

$$s^{*}=\frac{m^{*}}{\sum_{j=1}^{n}m_{j}},{\;m}^{*}=median\left\{\frac{x_{i}^{*}}{{\left(\prod_{j=1}^{n}x_{ij}\right)}^{1/n}}\right\},$$

where *x* denotes a p × n RNA-seq gene expression data matrix, with p genes and n samples. Let *xij* be the elements of RNA sequencing count matrix for *i*-th gene (i = 1, 2, . . ., p) and *j*-th sample (*j* = 1, 2, . . ., *n*). *m* ^*^ and *s* ^*^ are estimated using the geometric means derived from the training data.

Following normalization and log10-transformation, the RAS values for the GSL metabolism were calculated as previously described. The TP for each sample was then calculated and employed to adjust the RAS accordingly. Subsequently, the *ab initio* defined reactions were aggregated into GD2r+ and GD2r- variables for both the adjusted and unadjusted RAS datasets, enabling the prediction of decision values through the respective SVM model. These decision values provided quantitative estimates of the GD2 score across the test datasets.

### 2.7 GD2 two-gene signature

The normalized log10-transformed gene expression of *B4GALNT1* and *ST8SIA1* was summed to calculate the two-gene signature, as it was defined in the work of Sorokin et al. (2020).

### 2.8 Identification of CNA associated with the GD2 score

Statistical analyses were conducted to evaluate the relationship between the copy number alteration (CNA) status of genes and the GD2 score across subgroups of TCGA SARC:

The processed CNA data for the TCGA project was downloaded from the cBioPortal platform (data was retrieved on 2025-01-22). CNA data and gene expression data were integrated by Patient ID and the intersection of common genes (annotated in both as HUGO gene symbols). The CNA status levels −2, −1, 0, 1, and 2 were renamed to homozygous deletion, hemizygous deletion, no change, gain, and high-level amplification. The following filtering criteria were applied to ensure robust statistical analysis: Samples with missing CNA status values were excluded from the analysis; A minimum of 5 samples was required per subgroup-gene-CNA status combination; Genes were included only if they exhibited more than one unique CNA status level per group.

### 2.9 Statistical tests

Wilcoxon rank-sum tests were performed to assess differences in GD2 scores (based on *ras*, *rasTP*, *rasTPrec*) and two-gene signatures for two groups. Cliffs’s delta was computed with the effsize package (version 0.8.1). For the differences across more than two groups, the Kruskal-Wallis test was used. Dunn’s test was applied for *post hoc* testing using the rstatix package (version 0.7.2).

Kruskal-Wallis tests were performed to assess differences in GD2 scores across CNA status levels for each gene within each subgroup. Analyses were conducted separately for each subgroup of interest. Eta-squared estimates (η^2^) for the Kruskal-Wallis tests were calculated using the rstatix package (version 0.7.2). To control for the false discovery rate (FDR) in the context of multiple hypothesis testing, p-values were adjusted using the Benjamini-Hochberg method (Benjamini and Hochberg, 1995).

Genes were considered statistically significant if 1) the adjusted p-value was less than 0.05 and 2) the η^2^ exceeded 0.06, indicating a moderate or strong association.

### 2.10 Experimental assessment of GD2 expression

Human NB cell lines (CHP-134 and SH-SY5Y) were cultured under standard conditions. A fresh clear cell sarcoma of the kidney (CCSK) tumor sample from a pediatric patient was also processed for primary cell isolation. Flow cytometry was used to quantify GD2 surface expression using fluorochrome-conjugated antibodies. Detailed protocols for cell culture, tumor dissociation, and flow cytometric analysis are provided in Supplementary Material and Figures, Sections 1.2–1.4.

### 2.11 R package GD2Viz development

To facilitate access to GD2 score computation and visualization, we developed GD2Viz, an R package containing an interactive web application built using R Shiny. The R package allows users to calculate GSL RAS, predict GD2 scores, and explore precomputed datasets in an intuitive graphical interface.

GD2Viz is implemented in R (version 4.4.2) and follows Bioconductor guidelines to ensure integration within the Bioconductor ecosystem. Specifically, GD2Viz is designed to be interoperable with Bioconductor data structures, including SummarizedExperiment objects, enabling the user to supply their own data as input not only in tab-separated text files but also as RDS files containing R objects. For data privacy reasons, we separated precomputed RAS scores of several public RNA-seq datasets from the main app repository into a separate private GitHub repository. This results in an extended online version of GD2Viz at http://shiny.imbei.uni-mainz.de:3838/GD2Viz, which allows the user to explore these datasets in more depth. Without the presence of the dataset repository, the respective app tabs are hidden, and the app allows the user only to analyze their own data. Additionally, the package ensures adherence to Bioconductor’s package structure, including unit testing, version control, and documentation standards, to promote long-term usability and maintenance. GD2Viz is available at https://github.com/arsenij-ust/GD2Viz, and https://arsenij-ust.github.io/GD2Viz/.

## 3 Results

Accurately predicting GD2 levels from transcriptomic data requires careful consideration of the underlying biosynthetic pathway. While genes such as *ST8SIA1* and *B4GALNT1* are directly involved in GD2 synthesis, others like *B3GALT4* facilitate the conversion of GD2 into downstream gangliosides, thereby diminishing its cellular abundance. Moreover, some enzymes, e.g. *B4GALNT1*, exhibit low substrate specificity and participate in multiple reactions within the ganglioside biosynthetic network. These complexities highlight the limitations of simple gene-level inference and underscore the need for a pathway-informed modeling approach.

In our computational workflow, we addressed these challenges by modeling GD2-targeted pathway activity to predict GD2 expression in tumor samples. Our model integrates gene expression with pathway topology by mapping gene expression to enzymatic reactions (RAS values) and by using curated GD2-promoting (GD2r+) and GD2-mitigating (GD2r-) reactions as predictive features. To account for enzyme promiscuity and substrate ambiguity within the ganglioside pathway, we applied and evaluated two RAS adjustment methods that incorporate graph-based transition probabilities.

The results section begins with an evaluation of different SVM kernel functions, followed by a detailed assessment of the predictive performance of the *ab initio* GD2r+/GD2r- reaction set compared to alternative reaction combinations. We then provide external validation using independent RNA-seq datasets, followed by an experimental validation of GD2 expression in clear cell sarcoma of the kidney. Further, we identified *B4GALNT1* amplification as a potential biomarker in sarcoma. Finally, we present GD2Viz, an R Shiny tool designed to simplify the application of our methodology for researchers and clinicians.

### 3.1 SVM kernel evaluation

To evaluate the impact of different kernel functions on classification performance between NB and NT, we trained in total of 34 SVM models on transition probability adjusted RAS data, using various kernels, including linear, radial basis function (RBF), polynomial, hyperbolic tangent, Laplace, Bessel, and ANOVA kernels. Each kernel was evaluated across relevant hyperparameters. Models were compared based on precision, recall, F1-score, balanced accuracy (BA), and the number of support vectors (SVs), with the later serving as a proxy for model complexity.

While the RBF and Laplace kernels achieved the highest BAs (up to 0.86), they did so at the cost of substantially increased model complexity, requiring over 900 and 5,000 SVs, respectively. This raises concerns about overfitting, especially given the low dimensionality of the input (two variables derived from the summed activity of GD2r+ and GD2r- reactions) and the class imbalance (136 NB vs. 7,548 NT samples). In contrast, the linear kernel provided a strong balance between performance (BA = 0.80, F1 = 0.75) and simplicity (174 SVs), and was uniquely suited for interpretability in the context of cumulative GD2r+ and GD2r- activity.

Given these considerations, we selected the linear SVM as the default model for GD2 score computation throughout the remainder of the study. Full kernel comparisons, performance metrics, and decision boundary visualizations are provided in Supplementary Figure 2 and Supplementary Table 1.

### 3.2 Evaluation of GD2r+ and GD2r- reaction sets

We initially defined a curated set of GD2-promoting (GD2r+: R05946, R05940) and GD2-mitigating (GD2r-: R05939, R05948, R05947, R05941) reactions as features for the SVM model (Figure 3). These reactions were selected based on the biological relevance to GD2 biosynthesis, being located in the direct proximity of GD2 within the ganglioside pathway. To address ambiguity in enzyme substrate specificity, we applied two adjustment methods to the RAS values, resulting in three reaction activity variations, namely *ras* (unadjusted RAS values), *rasTP*, and *rasTPrec* (transition probability adjusted RAS values).

Having established that a linear kernel provides a well-generalized decision boundary, we next assessed the predictive power of the *ab initio* reaction set across all three RAS variations to test the robustness and specificity of the feature selection, we compared this set against 16 alternative GD2r+/GD2r- combinations, designed by systematically varying upstream and downstream reactions in the KEGG ganglioside biosynthesis pathway. In addition, the *ab initio* model was subjected to a permutation test using 10,000 randomly assigned reaction labels from the GSL metabolism, allowing us to evaluate whether its performance significantly exceeded what could be expected by chance.

Model performance was benchmarked on the highly imbalanced training dataset (136 NB vs. 7,412 NT samples), using precision, recall, F1-score, and balanced accuracy (BA), along with ROC and precision-recall (PR) curves. While ROC curves are commonly reported, metrics like PR AUC, F1, and BA are more informative for imbalanced classification tasks.

**FIGURE 4:**
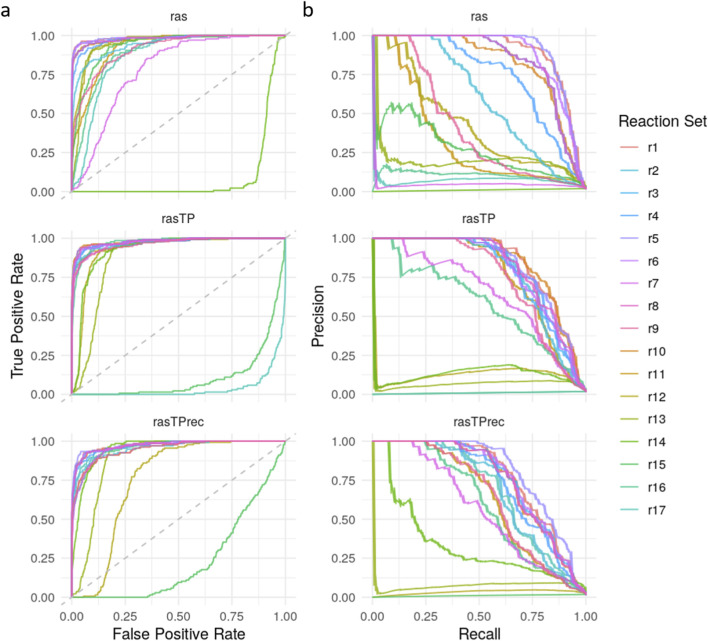
Performance comparison of GD2 prediction models using alternative reaction sets*.* **(a)** ROC curves and **(b)** Precision-Recall (PR) curves are shown for 17 different GD2r+ and GD2r- reaction set combinations (r1–r17, where r1 is the *ab initio* reaction set), evaluated across three RAS variations: *ras*, *rasTP*, and *rasTPrec*.

Figure 4 presents ROC and PR curves of all 17 reaction sets across the three RAS variants, with r1 representing the *ab initio* reaction set. A full table with detailed metrics for each model is provided in Supplementary Table 1. The r1 model demonstrates consistently strong classification performance across all RAS types. Balanced accuracy (BA) remains high for *ras* (0.85), *rasTP* (0.80), and *rasTPrec* (0.74), accompanied by correspondingly high PR AUC values of 0.90, 0.84, and 0.78, respectively. Overall accuracy (0.99) and specificity (1.00) are uniformly high across all variants, and the detection rate (0.01) and detection prevalence (0.02) are low, as expected due to the imbalanced dataset. The decreasing trend in recall from *ras* (0.70) to *rasTPrec* (0.47), paralleled by a similar decline in F1-score (0.80–0.63), suggests that the topological adjustment methods in *rasTP* and *rasTPrec* lead to increasingly conservative classification boundaries, reducing false positives at the expense of increased false negatives. These findings support the robustness of the *ab initio* reaction set and highlight the trade-off between sensitivity and specificity when incorporating pathway-informed topological constraints.

The results presented in Figure 4 and detailed in Supplementary Table 1 demonstrate that certain reaction sets maintain high classification performance across *ras*, *rasTP*, and *rasTPrec*, whereas others fail to distinguish between NB and NT samples effectively. As expected for imbalanced datasets, ROC curves tend to be overoptimistic, making PR AUC and balanced accuracy (BA) more reliable indicators of model performance.

Among all evaluated models, reaction sets r1 and r5 consistently exhibit the strongest classification performance, with r1 achieving BA values of 0.85, 0.80, and 0.74 and PR AUC values of 0.90, 0.84, and 0.78 across *ras*, *rasTP*, and *rasTPrec*, respectively. Reaction set r5 performs comparably, with BA values of 0.86, 0.79, and 0.78 and PR AUCs of 0.91, 0.83, and 0.81. Notably, r10 (in *rasTP*) and r6 (in *rasTPrec*) also show high performance. All high-performing sets share a common core: R05946 and R05940 as GD2r+ reactions and R05948 and R05947 as GD2r- reactions, emphasizing the importance of reactions immediately proximal to GD2 in the pathway.

In contrast, reaction sets r12 through r17 display near-random performance, with BA values around 0.5 and low PR AUCs across all RAS variants. Similarly, sets such as r2, r7, r9, and r11 show poor performance, particularly in *ras* and *rasTPrec*. These underperforming models either define only a single GD2r+ reaction (R05946 or R05940 alone), rely solely on R05948 as GD2r-, or incorporate reactions located too far upstream or downstream from GD2, undermining their ability to capture relevant biological signals.

To further assess the robustness of the *ab initio*-defined GD2r+ and GD2r- reactions, a permutation test with 10,000 random permutations of GSL reaction labels was performed. For each, an SVM with a linear kernel was trained on the *rasTP* values of the training dataset. The empirical p-values derived from these permutations were used to evaluate the significance of the model’s quality across BA, F1-score, precision, and recall. The results indicate strong classification ability, with a BA of 0.80 (p = 0.0006), F1-score of 0.75 (p < 0.0001), precision of 0.99 (p = 0.0007), and recall of 0.60 (p = 0.0006). These p-values suggest that the *ab initio* model significantly outperforms a random reaction classifier, providing strong evidence for its predictive capability.

Overall, the *ab initio* reaction set (r1) demonstrates consistently strong performance across all RAS variants, with high balanced accuracy and PR AUC, confirming its robustness and biological relevance. The results confirm that the *ab initio* GD2r+ and GD2r- reactions are robust classifiers with statistically significant predictive power. While alternative reaction combinations can maintain moderate classification performance, models lacking key reactions demonstrate a sharp decline in balanced accuracy.

### 3.3 Validation of the GD2 score on external datasets

**FIGURE 5:**
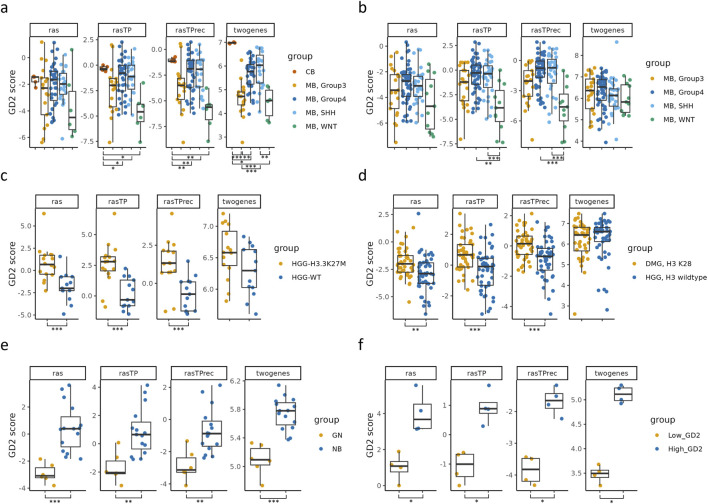
Validation of the predicted GD2 scores for six RNA-seq datasets and comparison with the two-gene signature. Boxplots visualizing the GD2 scores calculated on GD2r+ and GD2r- variables based on raw RAS, RAS adjusted by TP (*rasTP*), and recursive TP RAS adjustment (*rasTPrec*) values, as well as the two-gene signature, for six RNA-seq datasets, namely: **(a)** medulloblastoma (MB) subtypes and normal cerebellum samples (CB) from GSE203174. **(b)** medulloblastoma (MB) subtypes from the CBTTC cohort. **(c)** High-grade glioma (HGG) samples with an H3K27M alteration and H3-3 wildtype (WT) from GSE117446. **(d)** Diffuse midline glioma with an H3K27M alteration (H3K28 is a synonym for H3K27M) and high-grade glioma with H3-3A wildtype from the CBTTC cohort. **(e)** Ganglioneuroma (GN) and neuroblastoma (NB) from GSE147635. **(f)** Parental Kelly cells (neuroblastoma) stained with GD2-APC antibody and sorted in GD2-low and GD2-high populations from GSE180514. ^***^ = adj. p ≤ 0.001; ^**^ = 0.001 < adj. p ≤ 0.01; ^*^ = 0.01 < adj. p ≤ 0.05; not significant = adj. p > 0.05 (not illustrated). The sample numbers per group, p-values, and effect sizes are documented in Supplementary Table 3.

Next, we validated the GD2 score on six independent RNA-seq datasets, assessing its performance in distinguishing GD2 expression levels across cancer types in accordance with the literature’s expected expression (Figure 5 and Supplementary Table 3). Additionally, the GD2 score was compared to the two-gene signature published by Sorokin et al. (2020). The validation datasets included MB subtypes from GSE203174 and CBTTC datasets (Figures 5a,b), H3 wildtype and mutant high grade gliomas (HGG), and diffuse midline gliomas (DMG) (Figures 5c,d), GN and NB from GSE147635 (Figure 5e), and GD2-high/low parental Kelly cells (NB) stained with GD2-APC antibody from GSE180514 (Figure 5f).

In both MB datasets (Figures 5a,b), the subtypes exhibited distinct GD2 score distributions. The WNT subtype consistently displayed lower scores, while most of the samples of group 3, group 4, and SHH MB had significantly higher GD2 scores. This is in agreement with prior knowledge of GD2 expression patterns, where both SHH and group 4 express GD2, whereas group 3 is more diverse (Paret et al., 2022).

Samples harboring the H3.3K27M alteration in HGG and DMG (Figures 5c,d) had significantly higher GD2 scores than their H3 wildtype counterparts, supporting the hypothesis that GD2 expression is associated with this specific alteration (Mount et al., 2018; El Malki et al., 2023).

In Figure 5e, NB samples exhibited significantly higher GD2 scores than ganglioneuromas, aligning with the expected GD2 expression profile of these tumors (Paret et al., 2022; Wei et al., 2024). Similarly, the GD2 score confirmed a significant difference in GD2 scores between GD2-high and GD2-low NB Kelly cells.

The comparison between the GD2 score and the two-gene signature revealed significant differences in three of six datasets. In contrast to the GD2 score, the two-gene signature failed to capture the heterogeneous GD2 expression across MB subtypes in the CBTTC dataset and was similarly insensitive to differences between H3.3K27M mutant and H3 wildtype samples in HGG and DMG. In contrast, our GD2 score consistently reflected these expression differences, aligning with known biological variation. Both methods successfully distinguished between NB and GNB samples, as well as between GD2-low and GD2-high Kelly samples, demonstrating that the two-gene signature retains discriminatory power in clearly stratified contexts. Notably, while the two-gene signature indicated the highest GD2 expression in normal cerebellum (CB), exceeding that of MB tumor samples in the GSE203174 dataset, the GD2 score predicts a lower GD2 level. A further advantage of the GD2 score lies in its cross-dataset comparability. Indeed, thanks to the normalization of gene expression inputs during preprocessing, the GD2 score maintains interpretability and consistency across datasets.

Overall, these findings demonstrate that our method effectively captures biologically meaningful GD2-associated expression differences across multiple datasets and tumor types. While both the GD2 score and the two-gene signature perform similarly in clearly stratified samples, the GD2 score provides a more nuanced and consistent representation in complex and heterogeneous contexts. Notably, it avoids the biologically implausible overestimation of GD2 expression in normal brain tissues, such as the cerebellum, and more accurately reflects subtype-specific variation in MB, HGG, and DMG. These differences underscore the advantages of a pathway-informed, reaction-level approach over a minimal gene signature.

### 3.4 GD2 score evaluation of public transcriptome datasets accurately predicts GD2 expression in CCSK

**FIGURE 6:**
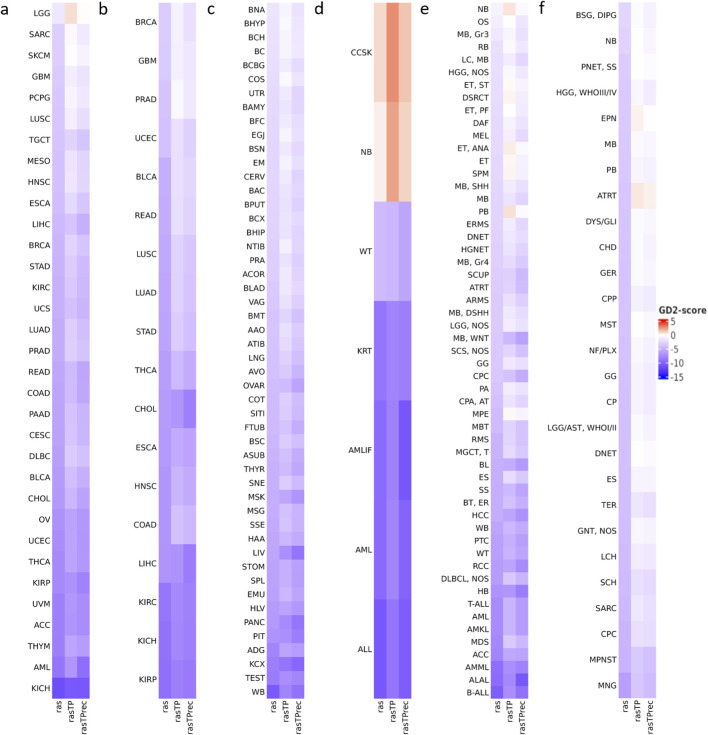
Variability of the predicted GD2 scores across cancer types for five RNA-seq datasets. The illustrated values are the median GD2 scores per cancer type and healthy human tissues. GD2 scores were calculated on GD2r+ and GD2r- variables based on raw RAS, RAS adjusted by TP (*rasTP*), and recursive TP RAS adjustment (*rasTPrec*) values. **(a)** TCGA tumor samples (including primary tumor, metastatic, and recurrent tumor sample types). **(b)** TCGA normal tissue samples. **(c)** GTEx normal tissue samples. **(d)** TARGET tumor samples. **(e)** St. Jude Cloud tumor samples. **(f)** Pediatric Brain Tumor Atlas CBTTC cohort tumor samples. The *ras*, *rasTP*, and *rasTPrec* values are comparable across the subfigures. Abbreviations can be found in Supplementary Table 2.

We used the GD2 score to investigate its distribution across various tumor entities using 5 transcriptome datasets. We computed the GD2 scores using the *ab initio* SVM model with a linear kernel. To further explore the impact of the RAS adjustment methods, the GD2 scores were computed from *ras*, *rasTP*, and *rasTPrec* values for each dataset. Figure 6 presents heatmaps of median GD2 scores for five RNA-seq datasets, grouped by cancer type or healthy human tissues: Figures 6a,b depict the TCGA tumor and normal tissue samples, respectively, while c, d, e, and f visualize the GTEx, TARGET, St. Jude Cloud, and the CBTTC from Pediatric Brain Tumor Atlas datasets. To make the values visually comparable between the heatmaps, the color scales were standardized across the subplots. The RAS variations are comparable across the datasets due to the size-factor normalization step applied between the training data and the used datasets during prediction.

Across TARGET, St. Jude Cloud, and CBTTC datasets, NB consistently exhibited very high GD2 scores in the median, as expected. Other pediatric tumors with known GD2 expression, such as medulloblastoma (MB) and diffuse intrinsic pontine glioma (DIPG), also showed elevated GD2 scores. Higher score was also found in ependymoma (ET/EPN) and high-grade glioma (HGG), which can express GD2 according to the literature (El Malki et al., 2023; Monje et al., 2025). Among pediatric sarcomas, expression is predicted in osteosarcoma (OS) and desmoplastic small round cell tumor (DSCRT), both entities with dismal prognosis. This is in accord with previously reported immune staining with anti-GD2 antibodies on frozen tissues (Dobrenkov et al., 2016).

Both TCGA and GTEx normal tissue samples generally exhibited low GD2 scores, reinforcing that GD2 expression is largely tumor-specific. Some brain-associated normal tissues from GTEx displayed higher GD2 scores than other normal tissues, but were notably lower than those of NB. The GD2r- values of normal brain tissue samples were found to be higher than those of NB, consistent with the fact that in normal brain tissue, simple gangliosides are metabolized into more complex species. In contrast, NB exhibits a stronger accumulation of GD2, indicating a distinct ganglioside metabolism (Supplementary Figure 3).

Notably, clear cell sarcoma of the kidney (CCSK), a rare and highly malignant neoplasm of childhood, also showed a remarkably high GD2 score. GD2 expression in this entity has not been previously described. To validate this prediction, we analyzed GD2 expression by flow cytometry in a CCSK tumor sample and compared the results with two NB cell lines.

**FIGURE 7:**
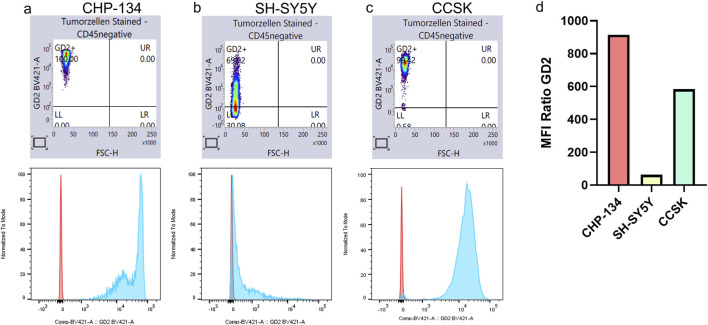
CCSK expresses high levels of GD2. GD2 expression was analyzed by flow cytometry in the two NB cell lines, CHP-134 **(a)** and SH-SY5Y **(b)**, and in one CCSK tumor sample **(c)**. In the lower panel, isotype control is shown in red and GD2 expression in blue. Panel **(d)** shows the ratio of mean fluorescence intensity (MFI) between the samples and the respective isotype control.

As illustrated in Figure 7, GD2 expression was detected in almost all CCSK tumor cells (99.4%), with an MFI greater than SH-SY5Y and more comparable to CHP-134, a cell line recognized for robust GD2 expression (Galassi et al., 2023). These results validate the accuracy of our scoring method in identifying GD2-positive tumors.

### 3.5 Intratumoral GD2 heterogeneity reveals B4GALNT1 amplification as possible biomarker for a GD2-positive sarcoma phenotype

**FIGURE 8:**
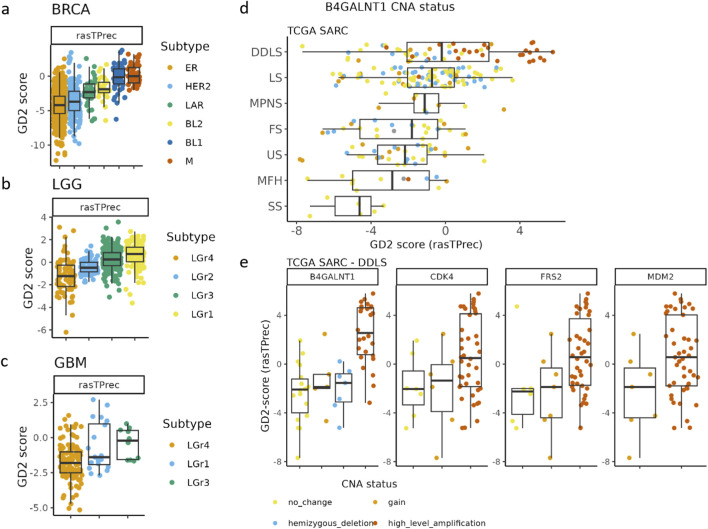
The GD2 score exhibits variability across breast cancer (BRCA), gliomas (LGG, GBM), and different subtypes of sarcoma (SARC). **(a)** TCGA BRCA mRNA-derived subgroups were assigned according to the reported results of Lehman et al. (2016). BRCA subtypes: ER, estrogen receptor; HER2 = human growth factor receptor 2; LAR = luminar androgen receptor; BL1 and BL2 = basal-like subtypes; M = mesenchymal. **(b)** TCGA glioblastoma multiforme (GBM), and **(c)** TCGA low-grade glioma (LGG). GBM and LGG subtypes were assigned based on reported gene expression clusters in Ceccarelli et al. (2016). **(d)** TCGA SARC grouped by subtypes and colored by the B4GALNT1 amplification status. DDLS = Dedifferentiated liposarcoma, LS = Leiomyosarcoma, MPNS = Malignant peripheral nerve sheath tumor, FS = Fibromyxosarcoma, US = Undifferentiated sarcoma, MFH = Malignant fibrous histiocytoma, SS = Synovial sarcoma. **(e)** CNA of *B4GALNT1*, *CDK4*, *FRS2*, and *MDM2* in TCGA DDLS samples.

Some TCGA tumor entities, such as gliomas (LGG, GBM), sarcoma (SARC), and some breast cancer subtypes (BRCA), exhibited median GD2 scores near the hyperplane, suggesting intratumoral heterogeneity in GD2 expression (Figures 8a–c). For further investigation, we retrieved molecular subtype information of these tumor entities by the TCGAbiolinks tool (Colaprico et al., 2016).

In BRCA, particularly the mesenchymal (M) and basal-like (BL1) subgroups exhibited the highest GD2 scores, followed by BL2 and LAR-positive subtypes. In contrast, estrogen receptor-positive (ER) and HER2-enriched subtypes showed in median lower GD2 scores but a higher variance across samples, suggesting that GD2 expression is enriched in basal-like and mesenchymal BRCA subtypes rather than luminal or hormone receptor-driven tumors.

GBM and LGG exhibited distinct mRNA-based subtype-dependent differences in GD2 scores. In GBM, LGr3 had the highest GD2 scores, while LGr4 had moderate to low scores. Notably, LGr1 has a mixed expression pattern with some samples having elevated GD2 scores. The GD2 score pattern across the mRNA subgroups of GBM is consistent with LGG (Figure 8c). Here, LGr1 and LGr3 exhibited higher GD2 scores compared to LGr2 and LGr4.

The GD2 score varied significantly across TCGA sarcoma subtypes, with the highest GD2 scores found in dedifferentiated liposarcoma (DDLS) (Figure 8d). In DDLS, the analysis revealed that CNA levels in *B4GALNT1* (adj. p-value = 0.02, and large effect size = 0.5) were significantly associated with differences in the GD2 score. Specifically, samples exhibiting high-level amplifications of *B4GALNT1* tended to have higher GD2 scores compared to those with copy number gain, no change, or deletion. These results are logical because *B4GALNT1*, located on chromosome 12q13.3, encodes one of the key enzymes in GD2 biosynthesis. In contrast, the adjusted p-values of commonly coamplified genes located on the same chromosome 12q13-15 *CDK4* (adj. p-value = 0.6, and moderate effect size = 0.07), *FRS2* (adj. p-value = 0.6, and moderate effect size = 0.09), and *MDM2* (adj. p-value = 0.6, and small effect size = 0.06) were not significant (Figure 8e).

Taken together, the GD2 score expression analysis highlights distinct GD2 expression patterns across various tumor and normal tissue types, with pediatric neuroectodermal tumors, sarcomas, and gliomas showing the highest GD2 scores. We detected a general absence of GD2 expression in normal tissues. Furthermore, the consistency of the GD2 score suggests that this approach is applicable across diverse transcriptomic datasets. The score may aid in identifying tumor types that have not previously been recognized as GD2-positive. Lastly, *B4GALNT1* amplification could represent a biomarker to identify GD2-positive sarcoma samples.

### 3.6 Simplifying the usage of the GD2 score methodology via the R shiny GD2Viz package

In order to facilitate access to the aforementioned methodology for scientists and physicians, we developed the GD2Viz R/Shiny package. This offers the possibility to carry out the necessary process steps, starting with the calculation of the RAS values for the GSL pathway from a non-normalized count matrix, up to the GD2 score prediction in one’s own R environment. The primary feature of GD2Viz is an interactive application that enables any researcher to effortlessly evaluate their data sets in terms of the GD2 score.

**FIGURE 9:**
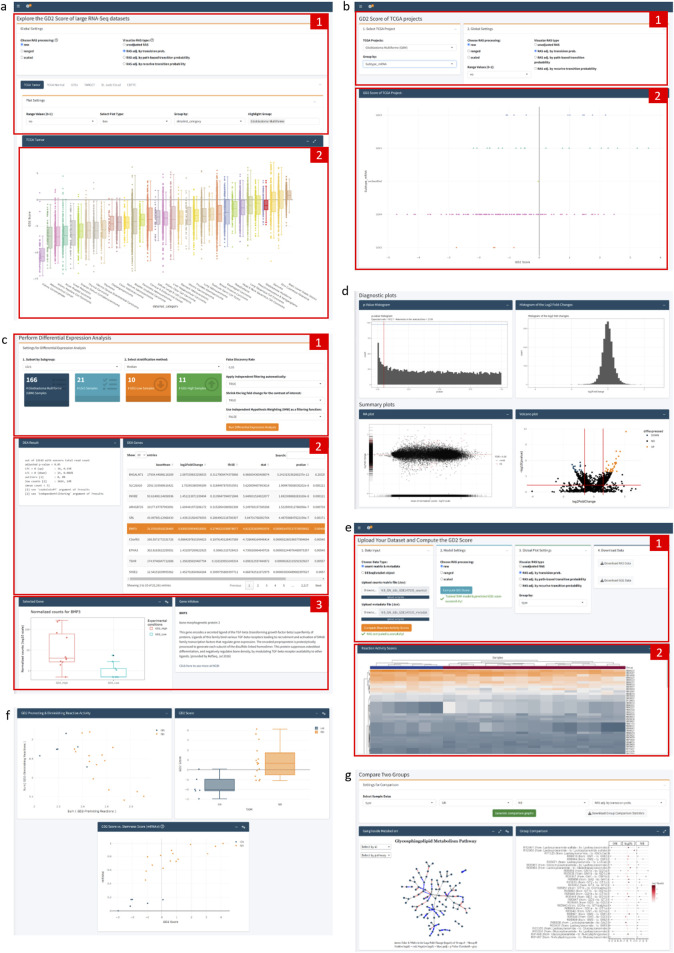
Annotated Screenshots of GD2Viz Shiny app. **(a)** First Tab contains the model and plot settings (1) and the GD2 score visualization of the selected dataset (2). **(b)** The top part of the second tab contains the selections for the TCGA project, the experimental variable, and the model settings (1). (2) shows the GD2 score of the TCGA project grouped by the selected molecular subtype. **(c)** Depicts the DEA part of the second tab, made up of the settings for the DEA (1), the summary of the DEA and statistics for each gene (2), and the gene expression and information of the selected gene (3). **(d)** Illustration of the diagnostic and summary plots of the DEA. **(e)** The top section of the third tab contains the file upload functionality, model and plot settings, as well as the possibility to download the computed RAS and GD2 score values (1). (2) shows the heatmap of the adjusted RAS. **(f)** Plots of GD2r+ and GD2r- variables, the GD2 score grouped by an experimental variable of the uploaded dataset, and the GD2 score against the stemness score. **(g)** Group comparison section of the third tab, visualizing the log2 fold-change of all GSL network reactions. Screenshots were taken of the GD2Viz app version 0.1.1.

The app consists of three tabs (Figure 9): The “Public Datasets” tab visualizes the precomputed GD2 score across six major RNAseq datasets: TCGA tumor samples; TCGA normal samples; GTEx; TARGET; St. Jude Cloud, and the CBTTC dataset from the Pediatric Brain Tumor Atlas. The user can choose between an interactive scatterplot, boxplot, or violin plot view, as well as explore the slightly different results by changing the SVM model settings, e.g. use raw, ranged, or scaled RAS values as data for model training or choose the preferred RAS adjustment method. Additional features, like grouping and highlighting by an experimental variable, allow further easy exploration of the datasets (Figure 9a).

The second tab focuses on single projects within the TCGA project (Figure 9b). It allows to analyze the individual tumor datasets in terms of clinical variables or molecular subtypes. Similar to the first tab, the model parameters can be selected. Besides the GD2 score visualization, the tab provides the possibility to run a differential expression analysis (DEA) for the entire project or a specific subset of samples (Figure 9c). For the DEA, the GD2 score is used for stratification of the samples in two (GD2-high vs. GD2-low) or three (GD2-high, GD2-medium, GD2-low) groups. The results display the overall result of the DEA, various diagnostic and summary plots, such as MA- and interactive volcano plots, a searchable table of significant genes, and additional gene information of the selected gene (Figures 9c,d).

The third tab enables users to upload their datasets and compute the RAS values and the GD2 score (Figure 9e). The input can be a raw count matrix, which is a commonly obtained object during RNA-seq analysis, and a metadata file as tab-separated values text file from an RNA-seq experiment or a DESeqDataSet object in .rds format. Users can compute adjusted and raw RAS values, change model settings, and predict the GD2 scores. Interactive visualization tools include adjustable heatmaps of RAS values, scatter plots showing GD2-promoting vs. GD2-diminishing reactions, and plots of the predicted GD2 scores. Additional plots illustrate the GD2 score against selected genes or stemness scores (Figure 9f). A comparison section provides a log2 fold-change analysis of Glycosphingolipid metabolism and a detailed ganglioside pathway analysis, including a network visualization (Figure 9g). The final section supports differential gene expression analysis based on GD2 score stratification, similar to the functionality of the second tab.

## 4 Discussion

Currently, 23 clinical studies (basket and entity-specific) are enrolling patients for GD2-directed therapies, including CAR-T cells, monoclonal antibodies, and antibody-drug conjugates (search term: https://clinicaltrials.gov/search?cond=GD2&aggFilters=status:rec, received on 2024-02-06). Evaluating GD2 expression using transcriptome data can help identify tumor entities with high GD2 levels and, given the considerable heterogeneity in GD2 expression, select suitable patients.

To predict GD2 expression based on transcriptomic data, we developed a computational pipeline integrating metabolic network modeling and machine learning. RAS were computed for the enzymatic reactions of the GSL metabolic network and adjusted by transition probabilities, incorporating the network topology. Using the cumulative RAS of different reaction sets containing GD2 mitigating and promoting reactions, an SVM was trained to distinguish neuroblastoma from normal tissue samples. We acknowledge that using neuroblastoma versus normal tissue as the training contrast risks capturing neural-crest lineage programs, which could bias predictions toward neural crest-derived tumors and reduce generalizability. We selected neuroblastoma as the primary comparison for training due to its clinical relevance and well-characterized ganglioside metabolism (Paret et al., 2025). The fundamental processes required for ganglioside biosynthesis, such as sphingolipid and ceramide synthesis, as well as lipid transport from the endoplasmic reticulum (ER) to the Golgi, are conserved across all cell types. The differences in ganglioside composition among tumors are primarily driven by the expression patterns of specific glycosyltransferases, which vary between tumor entities. However, our model does not consider competitive reactions entering lacto- or globo-series that may be relevant in other tumor entities, and therefore resulting in an overestimation of GD2 expression. Thus, our work should be viewed as a clinically motivated starting point in a well-studied entity with clear therapeutic impact. In the future this approach can be expanded and calibrated across additional tumor types by broaden the training dataset with multi-entity cohorts once paired GD2 ground truth is available.

The SVM model yields decision values rather than binary classifications. For a linear kernel in a two-class problem, these values correspond to the distance from the separating hyperplane and can thus be interpreted as a continuum. In the context of GD2 metabolism, the decision values provide a quantitative proxy for GD2 expression, capturing intermediate states between GD2-positive and negative phenotypes. The two model variables, being the sum of GD2r+ and GD2r-, further contextualize this continuum by indicating whether pathway activity favors GD2 accumulation (GD2r+ dominance) or its further metabolization (GD2r- dominance). While all evaluated models showed strong classification performance, the absence of direct GD2 expression measurements in the training dataset limits the ability to define a single best model. This underscores the need for experimental validation to establish the biological relevance of the GD2 score and support its clinical translation. Although a direct clinical translation is currently limited by the infeasibility of performing RNA-seq on each patient, our evaluation of the best-performing reaction sets has highlighted enzymes in close proximity to GD2 as potential biomarkers and motivates the development of curated panels assayed by targeted methods such as qRT-PCR or IHC. *ST8SIA1* and *B4GALNT1* promote GD2 formation, and have been utilized in several studies as a potential GD2 biomarker (Ruan and Lloyd, 1992; Lo Piccolo et al., 2001; Cheung et al., 2002; Mabe et al., 2022). Importantly, moving from gene-level readouts to reaction-level activity is essential, since enzyme promiscuity and substrate competition mean that single-gene measurements alone often fail to capture the biological state of GD2 metabolism. Future biomarker panels should therefore be designed around reaction-informed proxies, combining the expression of multiple enzymes into functionally meaningful readouts. Moreover, as tumor entities differ in their ganglioside composition, entity-adapted panels may be required. For instance, medulloblastoma group 3 is characterized by predominant GM3 expression, which in the presence of *ST8SIA1* and *B4GALNT1* can be converted to GD2 (Paret et al., 2022). In such cases, the joint analysis of these enzymes, even at the protein level by IHC, might be sufficient to stratify patients. A key challenge for clinical translation remains the determination of robust thresholds for biomarker positivity. Targeted assays currently lack large training datasets, which hampers the calibration of clinically meaningful cut-offs. Establishing such thresholds will require benchmarking against direct GD2 quantification methods and careful consideration of tumor-specific background metabolism.

With the rapid growth of advanced machine-learning methods for multi-omics integration, including deep learning architectures and pathway-aware models, there is understandable interest in leveraging these tools for biomarker discovery and prediction in oncology (Ballard et al., 2024; Zhang et al., 2025). However, several considerations make such approaches ill-suited for our specific objective and data regime. First, many deep learning methods require large, labeled training cohorts to avoid overfitting and to calibrate outputs. In this specific case, RNA-seq paired with quantitative GD2 measurements is currently unavailable at scale. Additionally, hyperparameter tuning is another challenge in small-data settings. The training dataset exhibits strong class imbalance, therefore in such imbalanced regimes, high-capacity models are prone to overfitting and minority-class bias without substantial additional tuning or data augmentation. Second, our predictor is intentionally low-dimensional. High-capacity models offer little upside in such a small feature space while increasing variance. Third, a central requirement within our study is interpretability: the ability to trace predictions back to specific enzymatic reactions and thereby identify biologically meaningful biomarkers. Deep learning and other ML methods typically yield non-linear sample-specific probabilities that are difficult to interpret in the context of pathway-informed biomarkers. By contrast, our linear SVM approach allows us to retain biological interpretability of the decision values directly corresponding to the balance of GD2-promoting versus GD2-mitigating reaction activity and demonstrated robust transfer across multiple independent datasets.

The GD2 score demonstrated advantages over the two-gene signature, particularly in capturing expression heterogeneity across tumor subtypes and maintaining consistency across independent datasets. Importantly, the score provides a continuous measure with a natural intermediate zone defined by the SVM hyperplane, facilitating interpretation of samples with ambiguous GD2 levels. In contrast, a two-gene signature lacks uniform scaling, requiring reference samples with known GD2 expression levels to contextualize results. Notably, our approach suggests low expression of GD2 in brain tissues, especially in the cerebellum, while the two-gene signature predicts high expression. A low amount of GD2 in the brain and peripheral nerves is in accord with clinical data showing no on-target off-tumor toxicity in patients treated with CAR-T cells against GD2 (Majzner et al., 2018; Straathof et al., 2020). This highlights the importance of incorporating GD2-diminishing reactions into the GD2 score. GD2 serves as an intermediate glycolipid in the synthesis of complex gangliosides characteristic of the mature brain, whereas simpler gangliosides like GD2 are more typical of the developing embryonic brain (Yu et al., 2009).

One limitation of our predictive model is the multi-layered regulation of GD2 expression, which is modulated by a number of factors, including the activity of key enzymes involved in ganglioside metabolism, namely the one encoded by *ST3GAL5*, *ST8SIA1*, *B4GALNT1*, and *B3GALT4*. The activity of these enzymes can be modulated by transcriptional and posttranslational mechanisms, including phosphorylation and N-glycosylation (Yu et al., 2004), and is further dependent on the developmental stage (Yu et al., 2009). These factors contribute to the variability of GD2 expression in normal and cancerous tissues, thus adding another layer of complexity to GD2 regulation. We acknowledge that the regulation of ganglioside metabolism is a complex process involving multiple factors that modulate the expression patterns and are not fully captured by our model. Another limitation is that the presence of a biosynthetic enzyme does not guarantee accumulation of its product. The pathway comprises opposing glycosyltransferases and glycanases operating in a tightly regulated, compartmentalized network, so flux depends on substrate availability, enzyme modifications, and competition among downstream reactions, which can decouple enzyme abundance from end-product levels (Allende et al., 2000; Kasprowicz et al., 2022). Nevertheless, previous research conducted on cell lines has demonstrated a general correlation between glycosyltransferase levels and the anticipated ganglioside content. Furthermore, a distinct correlation has been observed between multiple ganglioside synthase mRNA levels and the presence or absence of b-series gangliosides or complex gangliosides within the series (Ruan and Lloyd, 1992; Yoshida et al., 2001).

We confirmed high level of GD2 in CCSK, as predicted by the GD2 score, by measuring GD2 on a tumor sample by flow cytometry in comparison to NB. Notably, this is the first time that GD2 expression in CCSK has been demonstrated. CCSK is the second most prevalent pediatric kidney tumor, after Wilms’ tumor, and has a less favorable prognosis. New targeted approaches are urgently required to improve outcomes for patients with advanced-stage disease (Benedetti et al., 2024). Previous study suggests that CCSKs originate from a renal mesenchymal cell exhibiting a diverse array of neural markers. As a result, these cells appear prone to alterations also observed in various other neuroectodermal and neuronal tumors, which could explain the high level of GD2 expression (Cutcliffe et al., 2005). A limitation of our study is that GD2 expression was validated in a single CCSK sample; further investigations across larger cohorts are required to establish the prevalence and clinical significance of GD2 in this tumor type.

Using the RAS-based GD2 score, we were able to reproduce known data on GD2 expression across and within tumor entities. This includes heterogeneous expression of GD2 within different pediatric tumors such as NB, MB, and DMG. GD2 expression in NB varies among patient samples, with some exhibiting intermediate to low levels (Paret et al., 2022). This variability may be related to a more differentiated cell state (Paret et al., 2022). Our results align with these clinical observations, as some NB samples in both the training and test datasets exhibited intermediate to low GD2 scores. Heterogeneous and subtype-dependent GD2 expression in MB has been previously described (Paret et al., 2022). In accord with our results, the lowest expression of GD2 was detected in the WNT subtype, and the highest variability was in group 3, where some samples were GD2 negative due to the accumulation of GM3 (Paret et al., 2022). Further, our score indicates that in diffuse midline glioma, the H3K27M mutation is predictive of GD2 expression, as shown in previous work assessing GD2 expression by flow cytometry in pediatric high-grade gliomas with mutated or wild-type H3 (Mount et al., 2018).

Based on the GD2 score, heterogeneous GD2 expression can be expected in cancers with high clinical impact, such as GBM and breast cancer. In GBM, high GD2 expression has indeed been observed by using lipid analysis on tissue samples (Jennemann et al., 1990) but not all tumor samples expressed GD2. This underlines the necessity to assess GD2 expression before treating patients with anti-GD2 therapies or to identify biomarkers predicting GD2 expression. An anti-GD2 antibody-drug conjugate is currently in clinical testing for GD2-positive tumors, including glioblastoma (NCT06641908; first posted 2024-10-15).

In breast cancer, a GD2-positive population (up to 35%) has been identified by flow cytometry analysis of human breast cancer cell lines and patient samples. This GD2-positive population has a cancer stem cells phenotype and possesses the ability to form mammospheres and initiate tumors (Battula et al., 2012). Our data indicates that particularly two subtypes of triple-negative breast cancer (TNBC), the mesenchymal and the basal-like 1 subtype, express GD2. Expression of GD2 in TNBC has been previously described by immunohistochemistry of frozen samples and is associated with worse overall survival of patients (Ly et al., 2021). TNBC is very heterogeneous and is associated with high recurrence rates, distant metastasis, and unfavorable clinical outcomes. TNBC cells lack targetable receptors; hence, targetable markers are urgently needed. GD2 expression could be used to stratify these TNBC subtypes and as targets for therapeutic interventions.

Concerning the mechanisms related to the heterogeneous GD2 expression across tumor entities, the integration of CNA data into our analysis revealed a potential influence of *B4GALNT1* amplification on GD2 expression. *B4GALNT1* amplification was found particularly in some samples of DDLS. In DDLS, a significant correlation was observed between samples with high-level *B4GALNT1* amplification and elevated GD2 scores. The amplification of the 12q13-15 region, which includes key biomarker genes such as *MDM2*, *FRS2*, *CDK4*, as well as *B4GALNT1*, is a hallmark of DDLS (Dei Tos, 2000; Conyers et al., 2011; Jing et al., 2021; Nishio et al., 2021). As demonstrated in Figure 8e, not all samples with *CDK4* or *MDM2* amplification exhibit co-amplification of *B4GALNT1*. This suggests the existence of distinct breaking patterns within the 12q13-15 region, with one breakpoint potentially occurring between *B4GALNT1* and *MDM2*. Given that *B4GALNT1* is a key gene of the GD2r+ variable in our model, its overexpression or amplification (or that of *ST8SIA1*) may lead to higher GD2 scores, although the implicated GD2 overexpression and *B4GALNT1* amplification as a surrogate biomarker of GD2 overexpression require further validation.

## 5 Conclusion

Our study provides a refined understanding of GD2 expression across various tumor entities, highlighting its biological complexity and clinical implications. We present a novel computational framework for predicting GD2-positive phenotypes using pathway-adjusted reaction activity scores based on transcriptome data. We evaluated different SVM models and achieved robust GD2 score predictions across diverse datasets and cancer types.

H3K27M mutation in DMG was confirmed to be associated with elevated GD2 score, and CCSK was discovered and validated as a new entity with high GD2 expression. Further, *B4GALNT1* amplification was identified as a potential GD2-promoting factor in DDLS. Additionally, we developed the GD2Viz R package to facilitate broader adoption of our method. With our predictive GD2 score, we aim to provide insights into potential patient subgroups that could benefit from anti-GD2 therapy, underscoring the relevance of GD2 as a therapeutic target in ongoing and future clinical trials.

## Data Availability

The original contributions presented in the study are included in the article/Supplementary Material, further inquiries can be directed to the corresponding author. This study makes use of data generated by the following St. Jude Cloud projects: St. Jude Children’s Research Hospital – Washington University Pediatric Cancer Genome Project (PCGP) and Childhood Solid Tumor Network (CSTN) (Downing et al., 2012), St. Jude Children’s Research Hospital Genomes for Kids Study (G4K) (Newman et al., 2021), St. Jude Children’s Research Hospital Real-Time Clinical Genomics (RTCG) (Rusch et al., 2018), the Pan-Acute Lymphoblastic Leukemia Data Set of St. Jude Children’s Research Hospital (PanALL), and St. Jude Children’s Research Hospital Pediatric therapy-related myeloid neoplasm (tMN) Study (Schwartz et al., 2021). This study makes use of the UCSC Toil RNA-seq recompute compendium (Vivian et al., 2017). The results published here are in part based upon data generated by: the Therapeutically Applicable Research to Generate Effective Treatments (https://www.cancer.gov/ccg/research/genome-sequencing/target) initiative. The data used for this analysis are available at the Genomic Data Commons (https://portal.gdc.cancer.gov); the Genotype-Tissue Expression (GTEx) Project (https://www.gtexportal.org/); the TCGA Research Network (https://www.cancer.gov/tcga); and the Pediatric Brain Tumor Atlas: Children's Brain Tumor Tissue Consortium (CBTTC) (https://cbtn.org/) (Ijaz et al., 2020). The following datasets were used for validation and can be accessed via Gene Expression Omnibus (https://www.ncbi.nlm.nih.gov/geo/): GSE117446 (Krug et al., 2019), GSE147635 (Weiss et al., 2021), GSE180514 (Mabe et al., 2022). The code of our GD2Viz R package is available at https://github.com/arsenij-ust/GD2Viz. The documentation of GD2Viz can be found at https://arsenij-ust.github.io/GD2Viz/. The extended version of GD2Viz app can be found online at http://shiny.imbei.uni-mainz.de:3838/GD2Viz.

## Supplementary material

The Supplementary Material for this article can be found online at: https://www.frontiersin.org/articles/10.3389/fbinf.2025.1705930/full#supplementary-material

SUPPLEMENTARY FILE 1 Supplementary Material and Figures.

SUPPLEMENTARY TABLE 1 SVM models evaluation.

SUPPLEMENTARY TABLE 2 Abbreviations of cancer types and tissues of Figure 6.

SUPPLEMENTARY TABLE 3 GD2 score validation results.

## Glossary

AUC: Area under the curve
BA: Balanced accuracy
BL1/BL2: Basal-Like subtypes
BRCA: Breast cancer
CAR-T: Chimeric antigen receptor T-cell
CB: Cerebellum
CBTTC: Children’s Brain Tumor Tissue Consortium
CCSK: Clear cell sarcoma of the kidney
CNA: Copy number alteration
DEA: Differential expression analysis
DDLS: Dedifferentiated liposarcoma
DIPG: Diffuse intrinsic pontine glioma
DMG: Diffuse midline glioma
DSCRT: Desmoplastic small round cell tumor
ER: Estrogen receptor
ET/EPN: Ependymoma
FDR: False discovery rate
FFPE: Formalin-Fixed Paraffin-Embedded
GBM: Glioblastoma multiforme
GD2: Disialoganglioside
GD2r+: GD2 promoting reactions
GD2r-: GD2 mitigating reactions
GDC: Genomic Data Commons
GEO: Gene Expression Omnibus
GN: Ganglioneuroma
GPR: Gene-protein-reaction
GSL: Glycosphingolipid
GTEx: Genotype-Tissue Expression
HGG: High-grade glioma
IDH: Isocitrate dehydrogenase
IHC: Immunohistochemistry
KEGG: Kyoto Encyclopedia of Genes and Genomes
LC-MS: Liquid chromatography mass spectrometry
LGG: Low-grade glioma
MB: Medulloblastoma
NB: Neuroblastoma
NT: Normal Tissue
OS: Osteosarcoma
PR ROC: Precision-recall Receiver Operating Characteristic
RAS: Reaction Activity Score
RBF: Radial basis function
RNA-seq: RNA sequencing
SARC: Sarcoma
SHH: Sonic Hedgehog
SV: Support vector
SVM: Support Vector Machine
TARGET: Therapeutically Applicable Research to Generate Effective Treatments
TNBC: Triple-negative breast cancer
TP: Transition probability
