# Supplementary material for: Predicting GD2 expression across cancer types by the integration of pathway topology and transcriptome data
Source: Front Bioinform. 2025 Dec 4;5:1705930. doi: 10.3389/fbinf.2025.1705930 (PMC12711791; doi:10.3389/fbinf.2025.1705930)
Supplement: Supplementary file 3 [file Supplementaryfile1.docx]

Supplementary Material

**1. Supplementary Methods:**

**1.1. Data Acquisition and Preprocessing**

To model the continuous GD2 expression from low to high levels, NT and NB samples were used for the training dataset. In detail, the gene expression data (RSEM expected counts) and clinical information of the combined TCGA-TARGET-GTEx cohort (n=19,109) were obtained from the UCSC Xena database (<http://xena.ucsc.edu/>) (Goldman et al., 2020) (data was retrieved on 2022-10-06). This cohort is the result of the UCSC Toil RNA-seq recompute compendium (Vivian et al., 2017) and is available as log2(expected count + 1). The gene counts were subsequently transformed back to integer counts. The dataset was subset to neuroblastoma and normal tissue samples. Additional clinical information of the NB samples was derived from the Genomic Data Commons (GDC) Data Portal and merged according to the case submitter IDs (Grossman et al., 2016). Cell lines and ganglioneuroblastoma samples were removed. The final training dataset resulted in a total of 7,548 samples, of which 136 were NB and 7412 NT samples. The Ensembl IDs were annotated to gene symbols using the R package org.Hs.eg.db (version 3.14.0) (Carlson, 2017), resulting in 34,281 genes. The gene counts were normalized using the median of ratios method provided by the DESeq2 R package (version 1.34.0) (Love et al., 2014). Finally, a pseudocount was added and the counts were log10 transformed.

GD2 scores were predicted for the following publicly available datasets that were used for validation and subgroup exploration:

*TCGA*: The TCGA-TARGET-GTEx cohort was subset to TCGA project samples (n=10,529). TCGA molecular subtypes were retrieved using the TCGAbiolinks R package (version 2.22.4) (Colaprico et al., 2016). Additionally, molecular subtype information for TCGA breast cancer (BRCA) was retrieved as published in *Lehmann et al.* (Lehmann et al., 2016). For the cancer subtype analysis, the TCGA dataset was divided into subprojects. The Ensembl IDs were annotated to gene symbols. The copy number alteration (CNA) data of TCGA BRCA, glioblastoma multiforme (GBM), low-grade glioma (LGG), lung adenocarcinoma (LUAD), and sarcoma (SARC) was separately retrieved from the official cBioPortal for Cancer Genomics portal (<https://www.cbioportal.org/>) (data was retrieved on 2025-01-21).

*TARGET*: The TCGA-TARGET-GTEx cohort was subset to TARGET project samples (n=734). The Ensembl IDs were annotated to gene symbols. Tumor entities containing less than five samples per category were excluded. (Pugh et al., 2013)

*GTEx*: Similarly, the TCGA-TARGET-GTEx cohort was reduced to GTEx project samples. The Ensembl IDs were annotated to gene symbols. Tissue types containing less than five samples per category were excluded. Cell line samples were also excluded from the heatmap visualization resulting in a total of 7,845 samples. (GTEx Consortium, 2013)

*St. Jude Cloud*: RNA-seq profiles and corresponding annotation of 2,853 samples were obtained from the St. Jude Cloud (<https://stjude.cloud>) (data was retrieved on 2023-05-12). We excluded genes with less than 10 read counts in total of all samples. The batch effect caused by different library preparation protocols was removed using the ComBat_seq function from the sva R package (version 3.42.0). Tumor entities containing less than five samples per category were excluded.

*CBTTC*: The gene expression data and clinical information of the Pediatric Brain Tumor Atlas: Children's Brain Tumor Tissue Consortium (CBTTC) cohort (n=970) were obtained from the UCSC Xena database as log2(expected_count + 1) (data was retrieved on 2024-06-28). The gene counts were subsequently transformed back to integer counts (<https://cbttc.org>). (Ijaz et al., 2020)

*GSE117446*: Raw count matrix and genotype of 29 midline high-grade gliomas were retrieved from Gene Expression Omnibus (GEO accession: GSE117446). The dataset contained 16 H3K27M mutated and 13 H3 wildtype samples (data was retrieved on 2023-09-27). The dataset included the Ensembl gene IDs and gene symbols. The gene symbols were used in further analysis. (Krug et al., 2019)

*GSE147635*: Expression data and clinical information were obtained from Gene Expression Omnibus (GEO accession: GSE147635). The dataset contained 6 ganglioneuroma (GN) and 15 NB samples (Weiss et al., 2021) (data was retrieved on 2023-02-13). The processing of raw RNA-seq data for this dataset was described in our previous work (*Ustjanzew et al.* 2024 (Ustjanzew et al., 2024)).

*GSE180514*: Raw RNA-seq counts and GD2 status of 8 NB Kelly cells were retrieved from Gene Expression Omnibus (GEO accession: GSE180514). The dataset contained 4 GD2-low and 4 GD2-high samples, where GD2 level was identified by staining with GD2-APC antibody and FACS-sorted into the respective category. The Homo sapiens Annotation Release 109.20190905 was used for gene annotation (data was retrieved on 2023-11-21). (Mabe et al., 2022)

**1.2. Cell Culture**

Human neuroblastoma cell lines acquired from DMSZ (Braunschweig, Germany) were kept at 37°C and 5% CO₂ in 10 cm² cell culture dishes (SARSTEDT AG & Co. KG, Nümbrecht, Germany). CHP-134 cells were cultivated in RPMI-1640 medium (Sigma Life Science, St. Louis, USA), and SH-SY5Y cells were cultivated in Advanced DMEM (Sigma Life Science, St. Louis, USA). 1% penicillin/streptomycin (Dickinson and Company, Franklin Lakes, USA), 1% L-glutamine (Sigma Life Science, St. Louis, USA), and 10% FCS (Thermo Fisher Scientific GmbH, Schwerte, Germany) were added to each medium. Cells were split twice a week by a confluence of ~85%.

**1.3. Tumor Isolation**

A fresh tissue sample of the tumor was obtained for cell isolation. The cells of the tumor tissue were isolated under sterile conditions. The tissue was mechanically minced and enzymatically digested using 5000 μg/ μl liberase (Sigma-Aldrich, St.Louis, USA) and 240 Units of DNAse (Sigma-Aldrich, St.Louis, USA). After digestion, the cell suspension was filtered to remove debris and collected for further processing. This method ensures the recovery of viable tumor cells for subsequent analysis.

**1.4. Flow Cytometry**

350,000 cells were incubated with fluorochrome-conjugated antibodies targeting the relevant cell surface antigens, including CD45 (Becton Dickinson GmbH, Heidelberg, Germany), GD2 (Becton Dickinson GmbH, Heidelberg, Germany) and 7-AAD (Becton Dickinson GmbH, Heidelberg, Germany). Isotype controls IgG2a and IgG1 were used. Data analysis was conducted using the FlowJo software. CD45-negative cells served as the reference population to determine the GD2 content. The comparative data between the isotype controls and the stained samples were normalized to mode. The MFI ratio was calculated by dividing the MFI of the stained cells by the mean fluorescence intensity of the corresponding isotype control.

**References**

Carlson, M. (2017). org.Hs.eg.db. doi: 10.18129/B9.BIOC.ORG.HS.EG.DB

Colaprico, A., Silva, T. C., Olsen, C., Garofano, L., Cava, C., Garolini, D., et al. (2016). TCGAbiolinks: an R/Bioconductor package for integrative analysis of TCGA data. *Nucleic Acids Res.* 44, e71. doi: 10.1093/nar/gkv1507

Goldman, M. J., Craft, B., Hastie, M., Repečka, K., McDade, F., Kamath, A., et al. (2020). Visualizing and interpreting cancer genomics data via the Xena platform. *Nat. Biotechnol.* 38, 675–678. doi: 10.1038/s41587-020-0546-8

Grossman, R. L., Heath, A. P., Ferretti, V., Varmus, H. E., Lowy, D. R., Kibbe, W. A., et al. (2016). Toward a Shared Vision for Cancer Genomic Data. *N. Engl. J. Med.* 375, 1109–1112. doi: 10.1056/NEJMp1607591

GTEx Consortium (2013). The Genotype-Tissue Expression (GTEx) project. *Nat. Genet.* 45, 580–585. doi: 10.1038/ng.2653

Ijaz, H., Koptyra, M., Gaonkar, K. S., Rokita, J. L., Baubet, V. P., Tauhid, L., et al. (2020). Pediatric high-grade glioma resources from the Children’s Brain Tumor Tissue Consortium. *Neuro-Oncol.* 22, 163–165. doi: 10.1093/neuonc/noz192

Krug, B., De Jay, N., Harutyunyan, A. S., Deshmukh, S., Marchione, D. M., Guilhamon, P., et al. (2019). Pervasive H3K27 Acetylation Leads to ERV Expression and a Therapeutic Vulnerability in H3K27M Gliomas. *Cancer Cell* 35, 782-797.e8. doi: 10.1016/j.ccell.2019.04.004

Lehmann, B. D., Jovanović, B., Chen, X., Estrada, M. V., Johnson, K. N., Shyr, Y., et al. (2016). Refinement of Triple-Negative Breast Cancer Molecular Subtypes: Implications for Neoadjuvant Chemotherapy Selection. *PloS One* 11, e0157368. doi: 10.1371/journal.pone.0157368

Love, M. I., Huber, W., and Anders, S. (2014). Moderated estimation of fold change and dispersion for RNA-seq data with DESeq2. *Genome Biol.* 15, 550. doi: 10.1186/s13059-014-0550-8

Mabe, N. W., Huang, M., Dalton, G. N., Alexe, G., Schaefer, D. A., Geraghty, A. C., et al. (2022). Transition to a mesenchymal state in neuroblastoma confers resistance to anti-GD2 antibody via reduced expression of ST8SIA1. *Nat. Cancer* 3, 976–993. doi: 10.1038/s43018-022-00405-x

Pugh, T. J., Morozova, O., Attiyeh, E. F., Asgharzadeh, S., Wei, J. S., Auclair, D., et al. (2013). The genetic landscape of high-risk neuroblastoma. *Nat. Genet.* 45, 279–284. doi: 10.1038/ng.2529

Ustjanzew, A., Nedwed, A. S., Sandhoff, R., Faber, J., Marini, F., and Paret, C. (2024). Unraveling the glycosphingolipid metabolism by leveraging transcriptome-weighted network analysis on neuroblastic tumors. *Cancer Metab.* 12, 29. doi: 10.1186/s40170-024-00358-y

Vivian, J., Rao, A. A., Nothaft, F. A., Ketchum, C., Armstrong, J., Novak, A., et al. (2017). Toil enables reproducible, open source, big biomedical data analyses. *Nat. Biotechnol.* 35, 314–316. doi: 10.1038/nbt.3772

Weiss, T., Taschner-Mandl, S., Janker, L., Bileck, A., Rifatbegovic, F., Kromp, F., et al. (2021). Schwann cell plasticity regulates neuroblastic tumor cell differentiation via epidermal growth factor-like protein 8. *Nat. Commun.* 12, 1624. doi: 10.1038/s41467-021-21859-0

**2. Supplementary Figures:**

**
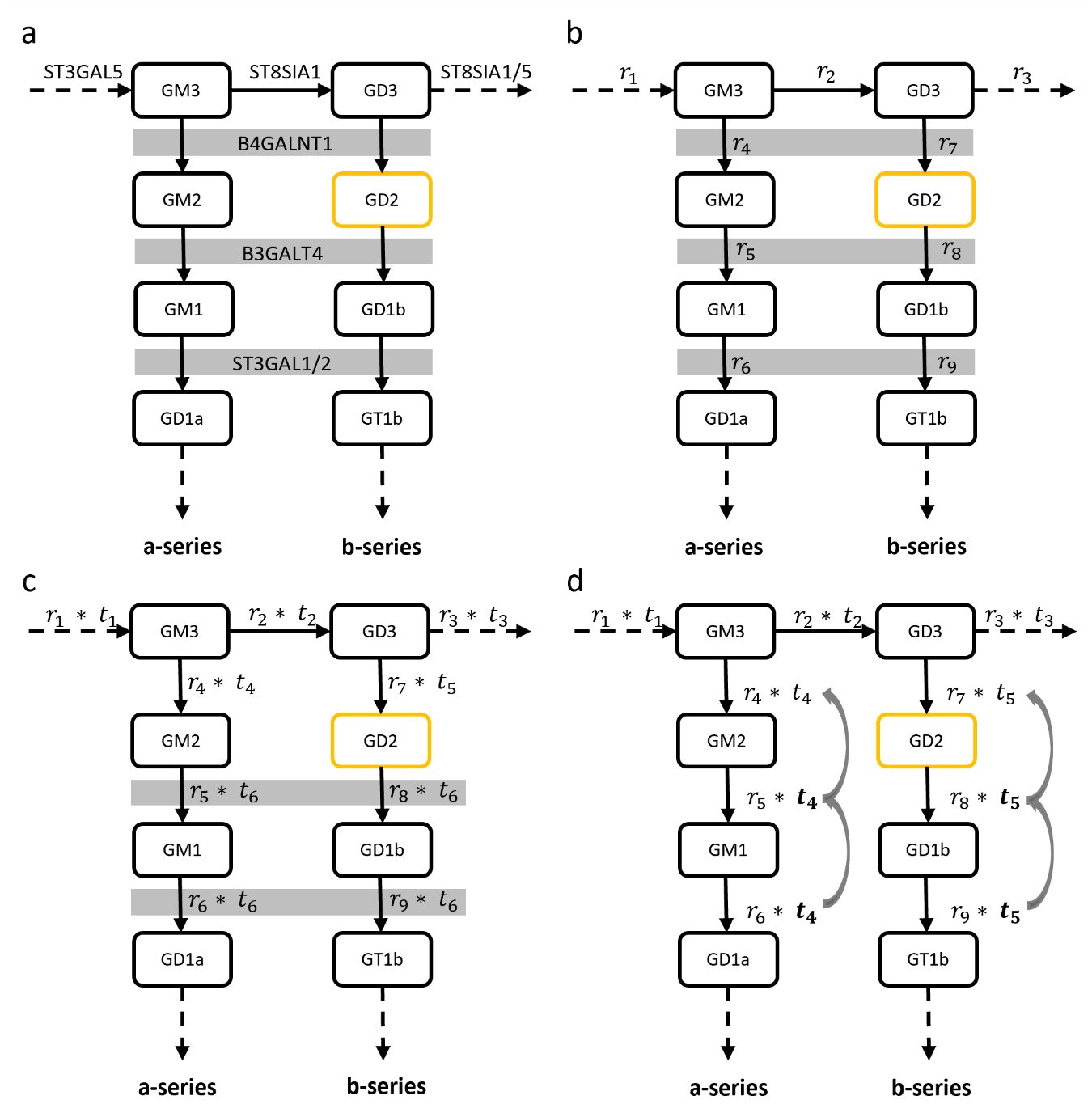
**

**Supplementary Figure 1. Mitigating enzyme promiscuity by integration of transition probability (TP) using the example of the sub-pathway of ganglioside biosynthesis.** a) a- and b-series of the ganglioside biosynthesis pathway are shown, with the genes encoding the involved enzymes represented by arrays and the metabolites by nodes. Gray bars indicate identical gene expression values across the series due to enzyme promiscuity. b) Computation of RAS values per reaction ($r_{n}$). Grey bars indicate identical RAS values across the series. c) Integration of TP values ($t_{n}$) resolves the initial reactions in the series. However, TP $t_{6} = 1$, meaning that downstream reactions share identical values across the series (grey bars). d) TP values equal to 1 (previously $t_{6}$) are recursively replaced by the first non-1 TP values occurring upstream in the pathway, finally completely resolving identical values across the series.

**
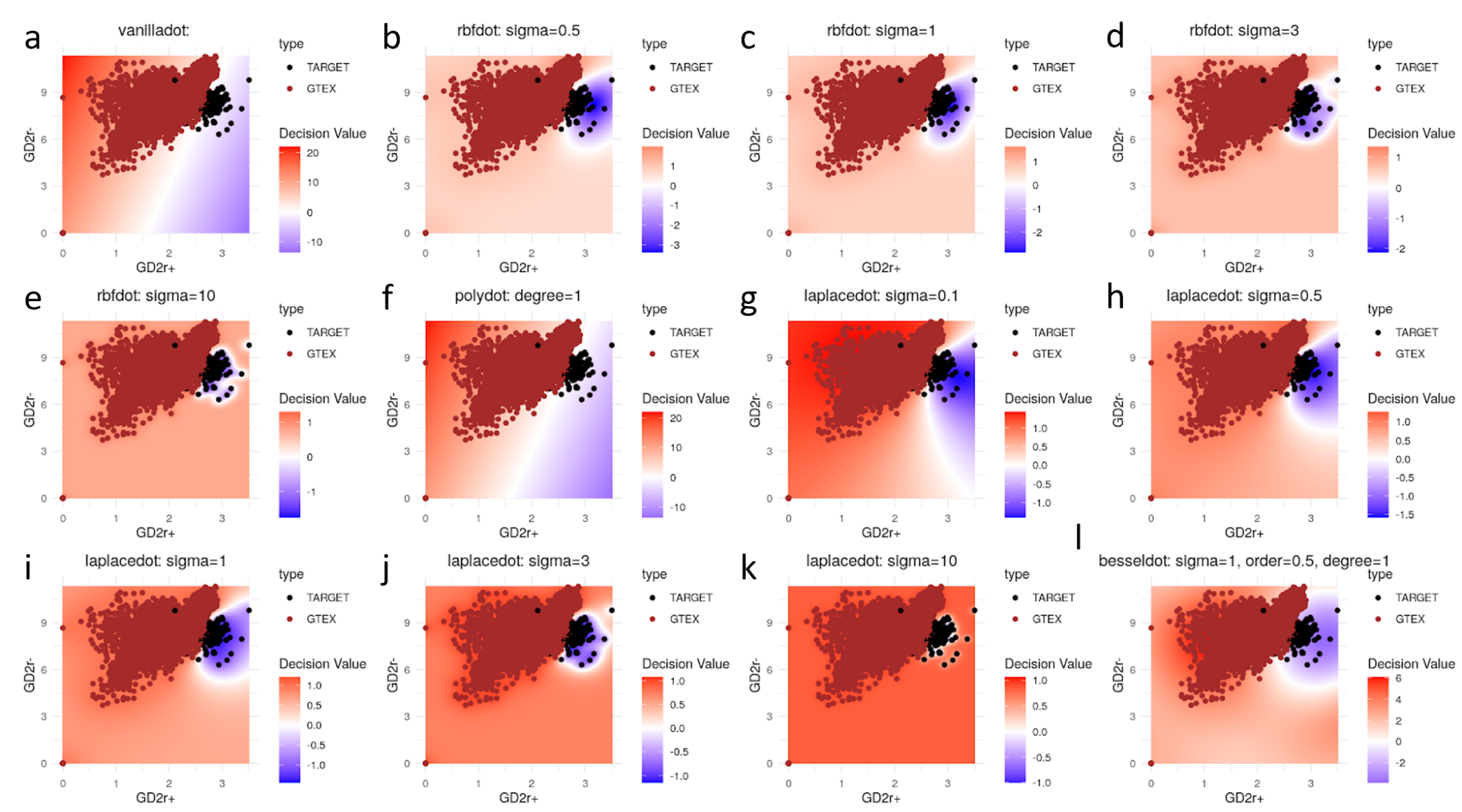
Supplementary Figure 2. Top 12 SVM models with the highest Balanced Accuracy on TP adjusted RAS training dataset.** The white area indicates the SVM hyperplane along the GD2r+ (x-axis) and GD2r- (y-axis) variables. Vanilladot: linear kernel, rbfdot: radial basis, polydot: polynomial, tanhdot: hyperbolic tangent, laplacedot: Laplace kernel, besseldot: Bessel kernel, and anovadot: ANOVA kernel.

**
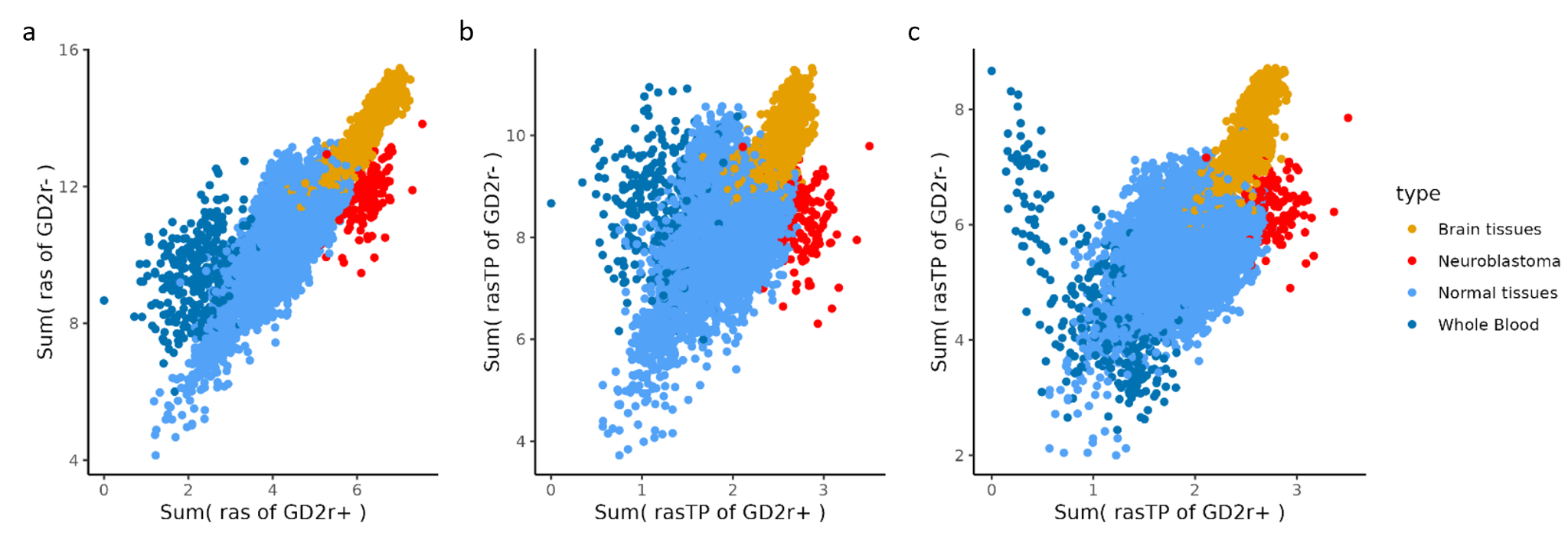
**

**Supplementary Figure 3. Scatterplots of the training dataset showing GD2r+ and GD2r- variables based on a) *ras*, b) *rasTP*, and c) *rasTPrec* values**.

**3. Supplementary Tables:**

**Supplementary File 1) (*Supplemental_file_1.xlsx*) SVM performance results of models with different kernels. Model performance metrics for different combinations of GD2r+ and GD2r- reactions calculated on the TP-adjusted RAS values of the training dataset.** #SV = Number of support vectors, Prec. = Precision, BA = Balanced Accuracy, rbfdot = Radial Basis kernel "Gaussian", polydot = Polynomial kernel, vanilladot = Linear kernel, tanhdot = Hyperbolic tangent kernel, laplacedot = Laplacian kernel, besseldot = Bessel kernel, anovadot = ANOVA RBF kernel.

**Supplementary File 2) (*Supplemental_file_2.xlsx*) Abbreviations of cancer types and tissues used for the heatmap visualization in Figure 6.**

**Supplementary File 3) (*Supplemental_file_3.xlsx*) Statistical results for 6 datasets used for GD2 score validation.** The file contains the sample size of groups, p-values, effect sizes, and 95%CI for GD2 scores based on *ras*, *rasTP*, *rasTPrec* per dataset. The results of the Kruskal-Wallis test and the post-hoc Dunn’s test were also reported for datasets with more than two groups.
